# Gene Therapy Approaches for the Treatment of Hemophilia B

**DOI:** 10.3390/ijms241310766

**Published:** 2023-06-28

**Authors:** Anastasiia B. Soroka, Sofya G. Feoktistova, Olga N. Mityaeva, Pavel Y. Volchkov

**Affiliations:** Life Sciences Research Center, Moscow Institute of Physics and Technology, National Research University, 141700 Dolgoprudniy, Russia; soroka.a.bio@gmail.com (A.B.S.); sayfutdinovas@gmail.com (S.G.F.); vpwwww@gmail.com (P.Y.V.)

**Keywords:** hemophilia B, gene therapy, AAV, genome editing, blood coagulation factor IX, hemostasis

## Abstract

In contrast to the standard enzyme-replacement therapy, administered from once per 7–14 days to 2–3 times a week in patients with severe hemophilia B, as a result of a single injection, gene therapy can restore F9 gene expression and maintain it for a prolonged time. In clinical research, the approach of delivering a functional copy of a gene using adeno-associated viral (AAV) vectors is widely used. The scientific community is actively researching possible modifications to improve delivery efficiency and expression. In preclinical studies, the possibility of genome editing using CRISPR/Cas9 technology for the treatment of hemophilia B is also being actively studied.

## 1. Introduction

Hemophilia B is a rare X-linked recessive hereditary disease of the hemostasis system resulting from abnormalities in the *F9* gene, which codes for blood coagulation factor IX and is located on the long arm of the X chromosome. Blood coagulation factor IX, also called Christmas factor, is a serine protease proenzyme involved in the blood coagulation cascade and dependent on vitamin K. Deficiency of the factor leads to prolonged bleeding that occurs spontaneously or after injury. The incidence of hemophilia B in the world is traditionally estimated at 1 in 30,000 male births worldwide, as males are the most affected, and females serve as carriers [1]. Nevertheless, there is growing recognition that women can also experience symptoms of hemophilia. While the severe form in women is rare, 25% of patients with mild hemophilia B in the U.S. hemophilia-treatment centers are women [2].

The severity of the disease usually correlates with the level of factor IX activity in the blood plasma. In mild hemophilia (>5% factor IX activity, >0.05 IU/mL), spontaneous bleeding is absent, but increased bleeding is observed after injuries and surgical operations. In moderate cases (1–5% activity, 0.01–0.05 IU/mL), spontaneous episodes of hemorrhage are rare, but even minor injuries provoke prolonged bleeding, and in severe cases (<1% activity, <0.01 IU/mL), spontaneous bleeding, hemorrhages in soft tissues or joints, and severe subcutaneous hematomas occur. Notably, patients with severe hemophilia account for 30–40% of all diagnosed cases of hemophilia B, according to the CDC [3]. 

In clinical practice, substitution therapy is used, which involves the intravenous administration of standard factor IX, obtained either from donor plasma or through recombination, once a week for bleeding or as prophylaxis and 2–3 times a week for severe hemophilia. Extended half-life factor IX products offer the advantage of reducing the frequency of administration up to once every 7–14 days [4]. Clotting factor IX concentrates obtained from human plasma, recombinant concentrates, and recombinant concentrates with an extended half-life, in which factor IX is combined with proteins (IgG1 Fc or albumin) or chemicals (for example, polyethylene glycol), are used [5,6]. 

Current prophylaxis treatment carries a notable economic burden to healthcare systems. In the CHESS II study, it was estimated that in the European Union, a mean annual total hemophilia-related direct medical cost per patient was EUR 235,723 [7], while in the CHESS US+ study, it reached USD 614,886 [8]. 

Another treatment for hemophilia is liver transplantation, which completely eliminates the symptoms of the disease [9]. However, this intervention is radical and carries the risk of serious adverse reactions.

Currently, alternative therapeutic approaches are being developed: recombinant proteins with an extended half-life, monoclonal antibodies directed to tissue factor pathway inhibitor (TFPI) [10], systemic delivery of *F9* mRNA [11], antithrombin-specific small interfering RNAs [12], and various gene therapy options with delivery of the *F9* gene or other factors (FVII or FV) of the coagulation pathway, and genome editing of the *F9* gene.

Gene therapy is a promising direction, as it can potentially provide long-term expression of factor IX after a single injection. For patients with severe disease, expression of factor IX as low as 5% will prevent spontaneous bleeding episodes and significantly improve quality of life. Among the possibilities for hemophilia B gene therapy, the following approaches can be distinguished: (1) correction of a defective gene copy using in vivo or ex vivo genome-editing tools, (2) control of protein translation without affecting the gene sequence using RNA interference, and (3) delivery of a functional gene copy of the *F9* gene with the help of viral vectors in vivo or ex vivo in autologous cells of the patient, with subsequent transplantation.

However, gene therapy has so far been used only for a small number of diseases. An in vivo editing approach is being studied in two clinical trials for the treatment of Leber’s amaurosis (EDIT-101, NCT03872479) and hereditary transthyretin amyloidosis (NTLA-2001, NCT04601051). The ex vivo approach is more common, including the possibility of CRISPR/Cas editing of B-lymphocytes for CAR-T therapy of various types of oncohematological diseases (NCT04037566, NCT04637763), hematopoietic stem cells in sickle cell anemia (CTX001, NCT05477563), beta-thalassemia (BRL-101, NCT05577312) and HIV infection, and pancreatic endodermal cell editing in type 1 diabetes (NCT05210530). In the case of hemophilia B, genome editing is used mainly at the stage of preclinical studies.

A drug based on RNA interference for the treatment of hemophilia B, Fitusiran (ALN-AT3), has been developed and is currently in phase 3 clinical trials. The FDA has approved four small interfering RNA drugs for the treatment of rare metabolic disorders (Patisiran, Givosiran, Lumasiran, and Inclisiran).

The vast majority of clinical studies on hemophilia B gene therapy are focused on the systemic delivery of a functional copy of the *F9* gene using AAV vectors. AAV vectors have low immunogenicity, generally do not integrate into the genome, and more often form an extrachromosomal structure, the episome, which remains in the cell for a long time. These advantages of AAV make this delivery system promising for the development of gene therapies, as evidenced by the growing number of approved drugs and drugs in clinical trials [13,14].

Luxturna (Voretigene neparvovec, Spark Therapeutics, Philadelphia, PA, USA) and Zolgensma (Onasemnogene abeparvovec, Novartis Gene Therapies, Bannockburn, IL, USA), gene therapies based on AAV, have been approved by the FDA for the treatment of Leber’s congenital amaurosis and spinal muscular atrophy, respectively. In August 2022, the first gene therapy for hemophilia A (Roctavian, BioMarin Pharmaceutical, San Rafael, CA, USA) was approved in the European Union. Later, in November 2022 an AAV-based drug (Hemgenix, from UniQure, Amsterdam, The Netherlands, CSL Behring, King of Prussia, PA, USA) was approved by the FDA for the treatment of hemophilia B, which has become the most expensive drug in the world. Furthermore, in February 2023, the European Commission granted conditional marketing authorization for Hemgenix.

In this review, we will consider in detail the features of the drugs under development for gene therapy of hemophilia B as well as the model objects used for their testing. 

## 2. Clinical Gene Therapy Studies

Clinicaltrials.gov lists 23 clinical trials of hemophilia B gene therapies (active and interrupted), of which most candidate drugs are AAV-based therapies, with the exception of a study involving lentiviral delivery of the *F9* gene to autologous hematopoietic and mesenchymal stem cells (NCT03961243; see Table 1).

The general approach in these studies is to intravenously administer an AAV vector with liver affinity carrying a transgene containing a functional copy of the *F9* gene under a liver-specific promoter. Codon optimization and elimination of immunogenic CpG motifs from expression cassettes are frequently utilized. Several clinical studies use *F9*-Padua, a variant of the *F9* gene with one amino acid substitution (R338L), which is 5–10 times more active than wild-type *F9* and was initially found in patients with thrombophilia [15]. Despite the recognition of *F9*-Padua as a game-changer in hemophilia B gene therapy, it is important to emphasize that there is a problem of assay discrepancies when evaluating post injection factor IX Padua levels. It should be taken into consideration that different assays can show varying factor IX activity values [16].

As of May 2023, two AAV-based drugs were in phase 3 clinical trials: SPK-9001 (PF-06838435/Fidanacogene elaparvovec, Pfizer, New York, NY, USA) and AMT-061 (Etranacogene dezaparvovec, UniQure), which received FDA approval.

The long-term follow-up results of patients who received an injection from phase 3 clinical trials indicate the safety and continued expression of the transgene. In Fidanacogene elaparvovec (Pfizer), the average level of factor IX activity after 5 years was 19.8% (in the first year, 25.4%) [17]. Four patients who underwent surgery had no observed excess bleeding. Fidanacogene elaparvovec is a liver-specific synthetic capsid (AAV-Spark100) AAV vector delivering a codon-optimized *F9*-Padua gene expressed under a liver-specific ApoE/hAAT promoter. It has been shown to be safe, with no severe side effects observed during phase 1/2 clinical trials and with three patients having non-drug-related side effects in follow-up. 

AMT-060 (UniQure), an AAV5 vector carrying wild-type codon-optimized *F9*, was initially less effective, with a factor activity of 5.2–7.5% in phase 2 clinical trials after 3 years following administration of the drug to 10 patients. However, after replacing wild-type *F9* with *F9*-Padua (AMT-061, UniQure), with the preservation of all other elements of the vector [18] in phase 3 clinical trials (HOPE-B, NCT03569891), the average factor IX activity 18 months after injection was 34.3%. A total of 52 of the 54 participants who received the injection stopped prophylactic substitution therapy; of the remaining two, one participant with a low response level had a high titer of neutralizing antibodies to AAV5, and the other received only a partial dose of the drug (10% of intended) due to an adverse event of hypersensitivity and continued prophylactic replacement therapy [19]. In November 2022, FDA approval was obtained for the use of this drug under the trade name Hemgenix.

The safety of AAV gene therapy for hemophilia B is also evidenced by the results of the longest follow-up (12–15 years) of four patients who received an injection of AAV2-hFIX (Avigen, Alameda, CA, USA): no serious side effects were detected, and patients did not develop a stable hepatotoxicity or hepatocellular carcinoma [20].

The use of highly active *F9*-Padua has significantly influenced the development of gene therapy for hemophilia B, with many companies making use of it. In general, its use is considered safe, but a case with severe side effects has been reported. In phase ½ clinical trials of FLT180A (Verbrinacogene setparvovec, Freeline, Hertfordshire, UK), one patient experienced thrombosis of an arteriovenous fistula associated with an increase in the level of factor IX activity up of to 310% at the 4th week after administration [21]. It should be noted that this patient had comorbidities (renal failure, arterial hypertension, and high body mass index). In all other patients, after 27 months, stable factor IX activity remained at a normal level: 51–78% (five patients) and 23–43% (three patients). Approximately 10% of adverse events were associated with FLT180a and 24% with immunosuppression. 

However, it is not always possible to achieve long-term stable activity of factor IX. In a study of AskBio009/BAX 335 (Takeda, Tokyo, Japan) based on a wild-type AAV8 where *F9*-Padua is used as a transgene, only one person achieved stable factor IX activity at 20% for 4 years, while the factor IX activity of the others disappeared after 5–11 weeks. The use of corticosteroids did not help to stabilize the loss of activity; the authors suggest that this may be due to CpG oligonucleotides introduced into the vector during codon optimization and activation of innate immunity. Three patients presented with serious side effects that were not associated with the drug and did not lead to death; the occurrence of thrombosis and inhibitors to factor IX was also not diagnosed [22].

Recombinant and modified AAV vectors continue to be developed and tested. In November and December 2022, studies of a new drug, ZS801, based on AAV with a synthetic capsid were registered in China (sponsored by the Institute of Hematology and Blood Diseases Hospital). In July 2022, the results of a phase 1 trial of BBM-H901 from the same sponsor based on AAV with a synthetic capsid carrying *F9*-Padua were published. The safety of the drug was shown in 12 patients with prophylactic use of glucocorticosteroids 1 year after administration. No serious side effects associated with BBM-H901 have been found [23]. The authors noted that the establishment of the therapeutic level of factor IX activity occurred faster than in other studies due to more efficient transduction of AAV hepatocytes with a synthetic capsid.

Several studies of AAV-based drugs that deliver a functional *F9* variant were terminated in 2021–2022, in particular, SHP648 (NCT04394286, Shire, Lexington, MA, USA) and DTX101 (NCT02618915, Ultragenyx Pharmaceutical, Novato, CA, USA), but not due to drug safety problems. The current status of trials of the therapy based on lentiviral delivery of *F9* gene to autologous hematopoietic and mesenchymal stem cells and their subsequent transplantation into a patient (YUVA-GT-F901, SGIMI, and NCT03961243) is also currently unknown. The summary of factor IX activity, administered doses, and treatment response to gene therapy drugs, based on published data, is presented in Table 2.

## 3. Hemophilia B Models

In preclinical studies, the efficacy and safety of a gene therapy drug is tested in model objects: in cell cultures, which evaluate the efficiency of transduction, the level of transgene expression, cytotoxicity, and in animal models, which evaluate biodistribution, tissue specificity, transgene expression, toxicity, and immunogenicity and enable dose-administration-method selection.

To test the vectors used to deliver a functional copy of a gene or editing tools, cell lines derived from the liver are used, particularly Huh7, PLC/PRF/5, and Hep3B, with the necessary mutations introduced through editing [29].

A more accurate model is iPSC-derived hepatocytes from hemophilia B patients. For example, for therapy testing, HB-iPSC (SXMUi001-A) with the c.223C>T mutation (p.R75X) was created [30]. Mouse ESCs with the same nonsense mutation were also obtained [31].

An even more accurate in vivo model that reproduces not only the cell type from the patient but also the effects at the level of intercellular interaction that exists in a real organ are 3D organoids. For example, there has been research into the differentiation of fibroblast-derived iPSCs derived from a patient with severe hemophilia B (mutation c.1297G>A) into hepatocytes with 3D organization, for which, compared with 2D culture, a higher level of expression of albumin, a marker of hepatocytes, was found [32]. It was also shown that 3D culturing compared to 2D allowed iPSCs to differentiate into fully functional hepatocytes capable of secreting factor IX with clotting activity, making organoids a more relevant model.

Hemophilia B has not been found in wild-type mice; therefore, different variants of transgenic mice are used to reproduce the pathology. For example, *F9*-knockout mice are used to test therapies with the delivery of a healthy copy of the gene, and humanized mice are used to test editing tools on human regions of the genome.

A popular mouse model with undetectable levels of mRNA and factor IX in blood plasma has a knockout for the *F9* gene (B6.129P2-F9tm1Dws, Jackson Laboratory, USA) [33]. Knock-in mice expressing various variants of factor IX are also frequently used, in particular, mice carrying human *F9* with a missense mutation found in patients with severe hemophilia B (R333Q-h*F9*) under the mouse *F9* promoter, mice with wild-type human *F9*-coding sequence, and mice with mutations in mouse *F9* (K5A in the Gla domain of *factor IX*). Notably, in mice with R333Q-h*F9*, the transcript and factor IX are expressed at a level of less than 1%, which is typical for patients with severe hemophilia B, and K5A mice have a mild disease phenotype.

All mouse models of hemophilia B are characterized by the absence of spontaneous bleeding. However, they die shortly due to blood loss after the tip of the tail is cut off. In knockout mice, when h*F9* is injected with AAV, antibodies are typically formed, while knock-in mice do not form them; therefore, the former are preferable for studying immune response to therapy and for testing therapies for the inhibitory form of the disease [34]. Immunodeficient *F9*-knockout mice (*Rag2*-KO, *IL2*-KO, *Fah*-KO, and *F9*-KO) were also created for transplantation of hepatocytes obtained from iPSCs of patients [35]. Studies on mice have limitations in terms of translatability of their results to humans. While mouse models are often used to study new therapies prior to human trials, the chance of translation to humans is not always high, which is not limited to hemophilia but is a general issue in translational research.

As an alternative animal model of hemophilia B, dogs with spontaneously acquired mutations that were further established during breeding are used: a Cairn terrier with a mutation leading to the amino acid substitution E379G and the absence of detectable factor IX; a Lhasa Apso with a 5 nt deletion 772–776 and a g.777C > T substitution, also without detectable factor IX and without development of inhibitors upon administration of canine factor IX; and a Labrador retriever with a complete *F9* deletion that developed inhibitors [36].

Using CRISPR/Cas9 and the somatic nuclear transfer method, a porcine model of hemophilia B with *F9* knockout (117 bp deletion in the 5’-UTR and exon 1) was also created, which is characterized by frequent episodes of spontaneous bleeding and joint damage [37]. In this model, it was shown that the insertion of h*F9* facilitated bleeding, which indicates the possibility of in situ replacement of a defective gene with a functional one.

Among primates, there are no known models of hemophilia B, but they are used to assess the level of transgene expression, the dose and safety of the vector, as well as the immune response to therapy in late preclinical studies since they are most similar to humans [38]. 

Although hemophilia B animal models are widely used in preclinical studies, they have some potential limitations, including differences in immune responses to AAV and tissue tropism of AAV vectors compared to humans and immune reactions to human factor IX.

## 4. Genome-Editing Studies

Various groups of researchers are actively studying the possibility of genome editing in vivo or ex vivo by editing progenitor cells of the organ of interest to obtain a lifelong therapeutic effect in hereditary diseases of renewable organs. Viral vectors are used not only to deliver a healthy copy of the gene (which acts as a template for homologous replacement) but also editing tools (CRISPR/Cas or zinc finger nuclease—ZFN).

During the early stages of preclinical in vivo genome-editing research for hemophilia B, hemophilic mice were subjected to the administration of the AAV6-h*F9* and mAlb-targeted AAV8-ZFNs. The outcome of this study revealed notably elevated levels of circulating human factor IX, reaching approximately 3000 ng/mL [39]. In 2022, the phase 1 clinical trial of SB-FIX-1501 (Sangamo, Richmond, CA, USA), which was the first to use ZFN to perform genome editing in a patient with hemophilia B, was terminated. In this study, functional *F9* was inserted into a safe albumin locus. The study involved one person, and no adverse side effects were found. However, expression of factor IX only reached 1.1%, and the patient had to continue with replacement therapy. Long-term follow-up of this patient is still ongoing, partly because there are few cases of in vivo editing of the human genome, and each of them is of interest (NCT04628871) [40].

CRISPR/Cas-based therapies for hemophilia B have only reached preclinical trials. The primary concern is potential off-target effects, especially with in vivo editing, so the use of the CRISPR/Cas system on patients will require constant monitoring for undesirable off-target effects. In this regard, ex vivo editing with subsequent transplantation of edited cells may be a safer and more controlled approach.

Viral vectors (based on AAV and adenoviruses) are typically used to deliver the editing system into the body. In 2019, a long-term restoration of the normal phenotype was shown after adenoviral delivery of the CRISPR/Cas9 system and a template for homologous recombination, with the aim of inserting a normal copy of the *F9* gene into a ROSA26 locus safe for insertion in hepatocytes in R333Q mice characterized by the expression of defective h*F9* and the absence of m*F9* [41]. The experimental group was injected with two AAV5 vectors (the first one with Cas and guides; the second one with the m*F9* matrix) at a concentration of 1:3; the control group received the same adenovirus with Cas but without guides. It was shown that the plasma concentration of mouse factor IX in the experimental group was significantly higher after 238 days than in the group without editing. The experimental group reached 10% factor IX activity: the same level as in mild hemophilia B. 

The previously mentioned therapy design had a number of disadvantages. Firstly, off-target integrations were found in two mice into the retrotransposon sequence and into the gamma-secretase-activating protein locus, presumably associated with the activation of other double-strand break-repair mechanisms. The authors suggest that the use of longer homology arms may improve the results of in vivo therapy. Secondly, the formation of antibodies against adenovirus particles and Cas9 nuclease (35 days after injection and up to at least 189 days) and activation of CD8+ and CD4+ cells against them but not against murine factor IX were detected. As a consequence, this therapy requires the preliminary formation of immune tolerance to Cas9 in patients. Thirdly, Cas9 DNA continued to be detected 245 days after injection, which is also a potential risk that must be eliminated before clinical use. The authors note that adenoviruses cannot be considered a successful vector for the delivery of gene therapy components due to the preexisting immunity to adenoviruses in the human population and the high immunogenicity of this vector. In another study, adenoviral delivery of CRISPR/Cas9 system components was compared to naked DNA construct delivery (plasmid with Cas9-2A-GFP and HDR donor in the form of ssODN or plasmid) using a hydrodynamic tail-vein injection to correct the Y371D mutation in the *F9* gene in mice with this mutation. Hemophilic mice injected with ssODN and dsDNA showed an HDR rate of 0.56% and 1.56%, respectively, which helped to correct hemostasis. Although adenovirus delivery showed a higher corrective efficacy, there was no therapeutic effect due to the strong effects of hepatotoxicity. An HDR rate or 0.56% of the *F9* gene alleles in mouse hepatocytes in donor ssODN and 1.56% in donor dsDNA contributed to the restoration of hemostasis in mice, while adenovirus delivery showed higher corrective efficacy, but there was no therapeutic effect due to the strong effects of hepatotoxicity [42].

The efficiency of CRISPR/Cas editing using homologous recombination in vivo or in cell lines between different studies is about 5% [43,44]. Therefore, to achieve effective knock-in, administration of high doses of AAV with Cas9 and donor DNA is usually required, which is associated with off-target risks and higher production costs. An alternative method is to edit through non-homologous end joining, which does not require the introduction of a vector with donor DNA. Insertion of *F9* into the 3’-UTR of the mouse albumin locus by non-homologous termination with the Cas9-delivered AAV2/8 vector has been shown to correct hemostasis in adult and neonatal m*F9*-knockout mice for at least 48 weeks. Germ cell editing did not occur, and off-target effects were not detected. The use of *F9*-Padua and the liver-specific LP1 promoter allowed a dose reduction of 10–100 times compared to other studies, which resulted in insignificant titers of anti-Cas and anti-AAV2/8 antibodies [45,46]. However, with this approach, insertions of h*F9* in the opposite direction and donor AAV without ires-h*F9* were observed, but this had a lower frequency and did not cause serious side effects [47].

As an alternative in vivo editing option, lipid nanoparticles have been used to deliver CRISPR/Cas with guides targeting the antithrombin gene. Affecting antithrombin expression, which is an endogenous negative regulator of thrombin generation, may improve blood clotting and relieve symptoms of hemophilia. When using lipid particles, no active off-targets, liver toxicity, and significant immune response to Cas9 were found. The use of lipid particles made it possible to reduce the time of CRISPR/Cas action as well as carry out repeated administration of the drug, unlike AAV [48]. A similar approach is already being used in clinical trials in patients with transthyretin (ATTR) amyloidosis (NCT04601051). LNP-CRISPR-Cas9 has been shown to effectively inhibit pathogenic TTR expression by approximately 90%, with mild side effects [49]. Application of lipid nanoparticles and AAV-based and other viral vectors for liver and non-liver delivery is also reviewed well by Raguram et al. [50]

Recently, a novel in vivo therapeutic strategy for hemophilia B was developed. It included a rebalancing approach (reduction the amount of antithrombin) and h*F9* gene knock-in in *Serpinc1* gene, using a hybrid system of LNP-packed CRISPR and AAV-packed h*F9*-donor templates. This approach provided approximately 1000 ng/mL factor IX and restored coagulation activity to a normal level. An advantage of LNP delivery of CRISPR is its short-term stability of approximately 1–4 days. On the contrary, the AAV vector acts as an episome, providing long-term expression, which may be undesirable in the case of CRISPR/Cas gene therapies, as it may lead to more off-target double-stranded breaks [51].

An ex vivo editing approach is also being explored, in particular, autologous transplantation of hepatocytes differentiated from patient iPSCs after CRISPR/Cas mutation correction. In iPSCs obtained from a patient with severe hemophilia (mutation g.31280G>A), a functional *F9* was inserted into a safe AAVS1 locus (AAV integration site in the first intron of the PPP1R12C gene on chromosome 19) with the help of CRISPR/Cas9 [32]. On day 11 of differentiation, *F9*-KO mice were transplanted with corrected hepatocytes, and restoration of the normal phenotype was observed. The authors suggest that this approach may be relevant for children with severe hemophilia B, as cells in their bodies are actively dividing, which can lead to loss of AAV. However, in another study, where two approaches were used to correct a defect in iPSCs obtained from a patient using CRISPR/Cas9, after knock-in of the functional *F9* gene and point mutation correction, it was noted that transplanted and corrected hepatocytes expressed less albumin and showed less recovery of factor IX levels (10–15% versus 10–50%) compared with transplanted human hepatocytes from cadaveric material (positive control) [35]. The limited therapeutic effect can be explained by the peculiarities of cultivation and the heterogeneity of the cell population inherent in the process of differentiation, which reduces the number of cells that can be transplanted. A brief summary of the advantages and disadvantages of genome-editing and gene transfer approaches is provided in Table 3.

## 5. Current Challenges and Limitations

### 5.1. Immune Response to Factor IX

The formation of inhibitors (antibodies to factor IX) is the most severe complication in the treatment of hemophilia B, which can occur both with standard enzyme replacement therapy and with the use of gene therapy approaches. During standard prophylaxis, antibodies to factor IX in different studies were reported in only 3–5% of patients with severe hemophilia B. However, one of the latest studies with prospective follow-up showed a higher cumulative inhibitor incidence, namely 9.3% at 75 days after exposure and 10.2% at 500 days after exposure [52]. While inhibitors do not affect the amount and location of bleeding, they significantly increase the risk of acute allergic reactions and death in such patients. There are currently no effective protocols of the induction of immune tolerance (IIT) for hemophilia B.

A high level of inhibitors in the blood of patients is a criterion for excluding patients from the sample of possible participants in clinical trials and future therapies. It has been shown that delivery of *F9* by AAV or a lentiviral vector can lead to the elimination of inhibitors and the subsequent establishment of factor IX expression at therapeutic levels [53].

As an alternative approach to IIT, the possibility of using genetically edited B cells is being studied. Specifically, a lentiviral vector with IgG-h*F9* was developed that targeted CD20-expressing B cells and prevented the development of inhibitory antibodies to factor IX in a mouse model of hemophilia B [54]. Resting human B-cell receptor specificity was achieved by mutating the envelope glycoproteins of the measles virus combined with lentivirus (MV-LV vector) and adding a single-chain variable fragment specific for hCD20.

To address the problem of antibodies to factor IX, oral immune therapy is also being developed to ameliorate IIT. The use of plant cells (lettuce or tobacco) producing CTB-hFIX by a patient may help prevent the formation of inhibitors and anaphylactic reactions during substitution therapy [55]. The plant’s thick cell walls keep antigens from being destroyed by acid in the stomach until the cells are destroyed by intestinal bacteria, followed by release of the antigen combined with a transmucosal transporter (CTB) for passage through the intestinal epithelium.

Another method to get around the inhibition problem is the development of alternative gene therapy approaches that do not involve delivery or editing of the *F9* gene. Fitusiran (ALN-AT3, Sanofi, Paris, France), which is based on RNA interference, has shown its effectiveness in patients with hemophilia A and B regardless of the presence of inhibitors. Fitusiran is a double-stranded small interfering RNA, with one strand binding a 23 nt region in the SERPINC1 gene after insertion into the RISC complex. This gene encodes antithrombin, a serine protease produced in hepatocytes and inactivating thrombin, factor FXa, and, to a lesser extent, factors FIXa, FXIa, and FXIIa [56]. Fitusiran, unlike other gene therapies, does not provide a long-term effect; however, in comparison with standard replacement therapy, its administration is required much less frequently, i.e., 1 time per month, and due to the peculiarities of the action of this drug, antithrombin activity decreases gradually and by day 30 reaches values of five times lower than normal.

Another option is using FVIIa in gene therapy for hemophilia B as a bypassing agent to promote blood clotting, as this factor can activate the coagulation cascade independently of factor IX [57] or FVa, which has the capacity to enhance the rate of thrombin generation almost 10,000 fold [58]. In hemophilia B mice, normal aPTT level was achieved over 28 weeks after AAV8/hFVa vector injection, and no risk of thrombosis has been shown. Otherwise, the FVIIa-based gene therapy led to thrombosis and premature mortality in hemophilia B mice. Thus, the minimal efficacy and the maximal safe level of such therapies should be assessed.

It has also been shown that in mice with preexisting immunity to factor IX, platelet-targeting therapy is effective, as they have the ability to absorb and accumulate plasma proteins in their alpha granules (including factor IX), which, when activated, are released. For example, in the work of Schroeder J.A. et al. (2021), Sca-1+ cells derived from hemophiliac mice were transduced with a codon-optimized *F9*-Padua lentivirus under a platelet-specific αIIb promoter and, after irradiation, transplanted into hemophiliac mice with anti-factor IX antibodies. It has been shown that, despite the presence of exogenous factor IX in this approach, platelet-specific therapy does not cause anaphylaxis and the formation of anti-FIX antibodies but, on the contrary, leads to the elimination of preexisting antibodies and can provide the production of factor IX after their elimination. Notably, the combination of a highly effective *F9*-Padua variant, irradiation, and a proteasome inhibitor accelerated the elimination of antibodies [59].

### 5.2. AAV Immune Response

A significant limitation of all AAV therapies is the presence of AAV-neutralizing antibodies in some patients who have had a previous infection—such patients are usually excluded from clinical trials. Various approaches are being explored to overcome immunity to the delivery vector, including plasmapheresis, the use of immunosuppressants, IgG proteases, CpG reduction, induction of regulatory T cells, capsid variant switching, the addition of empty capsids, and the creation of synthetic capsids [60].

The presence of neutralizing antibodies to AAV may not always lead to the absence of a therapeutic effect in gene therapy. In a study of AAV5-based AMT-060, a test with AAV5 reporter vectors with luciferase was retested for neutralizing antibodies more sensitive than the original test with AAV5 reporter vectors with GFP. The results showed the presence of antibodies to AAV5 in three of ten patients who were initially negative for anti-AAV5 neutralizing antibodies, and in two of them, an increase in the immune response was confirmed after administration of the AAV5-hFIX preparation. Despite this, no correlation was found between the level of *F9* expression and the level of antibodies since one patient had a low level of factor IX activity, and the second had the highest level of the low-dose cohort [61]. Therefore, in phase 3 clinical trials, patients with antibodies to AAV5 were not excluded. In the Etranacogene Dezaparvovec Hope-B clinical trial, participants with preexisting anti-AAV5 neutralizing antibodies exhibited 31.1% mean factor IX activity, while those without such antibodies had a mean factor IX activity level of 39.9% [19]. It was noted that the effect of neutralizing antibodies may differ for different serotypes, a feature of AAV5 that may be associated with high variability in the capsid sequence.

There is also the problem of the cross-reactivity of antibodies. For example, cross-reactive antibodies to the synthetic AAV-Spark100 (Pfizer) were associated with low expression of the h*F9* transgene, and such patients were also excluded from the study [25]. The presence of antibodies to wild-type AAV2 was shown 15 years after the introduction of the drug AAV2-hFIX (Avigen). At the same time, these antibodies also cross-reacted with wild-type AAV5 and AAV8 [20].

In addition to antibody formation, immunological rejection of transduced cells can also occur due to CD8+ directed against the AAV capsid. Therefore, if necessary, immunosuppressants such as prednisolone are used to suppress the occurrence of an immune response when high doses of the AAV vector are administered [62].

### 5.3. Disadvantages of AAV as a Delivery System

AAVs are characterized by low capacity compared to other viral vectors. For example, the capacity of a lentiviral vector is up to 9 kb, which is twice the capacity of AAV (4.7 kb). The capacity of the AAV vector is not an obstacle in the case of the delivery of a functional copy of the *F9* gene, the coding part of which is 1386 bp; however, during genome editing, when AAVs are used to deliver the CRISPR/Cas system, the capacity of the vector becomes critical.

AAV vectors are stored as an episome in the cell, so they are advantageously used in non-dividing or infrequently dividing cells rather than in cells of rapidly growing organs, such as the liver in children, where loss of transgene expression can occur. In such cases, integration into the genome of a functional *F9* sequence is an attractive approach. At the same time, since gene integration is often associated with the risks of insertional mutagenesis and subsequent oncogenesis, it can be advantageous to consider using more controlled approaches of editing, such as those based on the CRISPR/Cas system.

Genome editing is developing at a rapid pace, but its use in humans is still hampered by possible off-target effects. Even in the presence of mismatches in target sequences, Cas nucleases can still introduce double-strand breaks outside target regions; therefore, strategies are being developed to improve specificity and optimize CRISPR components [63]. In a study by He X. et al. (2022) performed in vivo, CRISPR/Cas9 editing in *F9* was performed in knockout mice via non-homologous end joining. As a result, deletions were found near the target sites, but no off-target editing was detected at the genomic and transcriptome levels, which means that risk minimization is possible with well-chosen guide RNAs and target sites [47]. Additionally, off-target reduction can potentially be achieved using engineering high-precision variants of Cas9, such as HFCas9, eCas9, HypaCas9, or SniperCas9 [64,65,66].

Another potential problem with genome editing is the insertion of AAVs, which deliver the CRISPR/Cas or ZFN system, into double-strand break sites instead of the transgene. One study showed a high frequency of such insertions (up to 47%) in mice after in vivo injection [67]. A comprehensive study of all possible consequences of genomic editing is necessary before its application at the stage of clinical trials.

## 6. Future Prospects

Currently, researchers are actively working on developing strategies to improve the effectiveness of gene therapy approaches. Particularly, the creation of synthetic capsids may help address the preexisting immune response to wild-type AAV serotypes. To date, the primary method for obtaining synthetic capsids is an in-vivo-directed evolution. This method involves the creation of libraries of capsids followed by selection according to specified criteria, for example, selection of capsids in the presence of human immunoglobulin and isolation from human hepatocytes transplanted into chimeric mice [68,69].

The use of *F9* gene variants with a higher coagulation efficiency of the expressed protein makes it possible to increase the effectiveness of the drug regardless of the chosen gene therapy strategy. The most popular naturally occurring sequence variant is *F9*-Padua. Synthetic variants of *F9* are also being developed, carrying various missense substitutions, for example, CB 2679d-GT with the three mutations R318Y, R338E, and R343R; F9-E456H with increased platelet specificity; and a number of other variants showing increased in vitro and in vivo activity in animals [57,70,71]. The use of highly efficient gene variants makes it possible to reduce the dose of the administered vector, which reduces the risk of hepatotoxicity but requires careful study of their safety, as such variants carry the risk of thrombosis when overexpressed. After injection of *F9*-Padua, one case of thrombosis was described in a patient with prothrombotic diseases [21].

To reduce the injected dose of the drug and increase its effectiveness, tissue-specific promoters are used, which makes it possible to limit the type of cells in which the transgene is expressed. In addition, the correct selection of the AAV serotype, which is relevant for the organ of interest, or the creation of a synthetic capsid with a given tropism can increase the specificity of delivery. The use of glycosylation and ubiquitin-like modifications such as SUMOylation and neddylation in AAV2 vectors increases the efficiency of transduction in vivo and increases the expression level of factor IX (up to 2-fold) in a mouse model of hemophilia B [72,73]. It has also been shown that FerA domains from proteins of the Ferlin family involved in vesicle fusion and membrane transport can directly interact with the surface of AAV, enhancing the ability of the capsid to bind to target cells. For example, systemic injection of scAAV8/hFIX-FerA increased *F9* expression 4-fold compared to scAAV8/hFIX and improved hemostasis in *F9*-knockout mice [74].

The advent of newer approaches of genome-editing techniques, such as base editing and prime editing, allows introduction of point mutations in the DNA without generating double-stranded breaks. Base editors can convert one DNA base to another; for example, cytidine base editors allow C > T conversions, and adenine base editors allow A > G conversions. Base editing using SpCas9-NG with a broad PAM flexibility has been applied for correction of point mutation (c.947T > C; I316T) in hemophilia B patient-derived iPSCs [75]. Compared to base editors, prime editors are more flexible and allow all possible base conversions, small insertions, deletions, as well as their combinations at target sites; thus, it can potentially be used to correct up to 89% of known genetic variants associated with human diseases [76]. While genome editing shows promise in treating certain diseases (e.g., sickle-cell disease [77]), its application in other cases can pose challenges due to a variety of mutations to target, e.g., in solid tumors. To date, there is a limited number of clinical trials involving genome-editing drugs, with small numbers of participants involved. The progress of ongoing research heavily relies on the outcomes of these trials.

## 7. Conclusions

Hemophilia B is a disease that can be completely cured by gene therapy, as it is caused by single defects in the *F9* gene and has a wide therapeutic window. In 2022, the first gene therapy for hemophilia B based on the AAV vector was approved. Hemgenix (UniQure, CSL Behring), an AAV5 vector delivering a functional, highly active copy of *F9*, has been shown to be effective and safe. However, accumulating data on modifications of AAV vectors that increase the efficiency of transgene delivery and expression as well as on the first successes in genome editing in model animals indicate that it is necessary to continue the development of “best in class” solutions. The use of editing based on CRISPR/Cas technology may be a promising approach, as it can provide long-term expression of *F9* in contrast to the delivery of a healthy copy using AAV, which may lose expression in the long term.

## Figures and Tables

**Table 1:** Clinical trials of gene therapies for the treatment of hemophilia B presented on the website clinicaltrials.gov, accessed on 15 May 2023.

Sponsor	Therapy	Capsid	Promoter	Transgene	Phase	Status	Identifier on clinicaltrials.gov
UniQure, CSL Behring	AMT-060	ssAAV5	Liver-specific	*F9*	1/2	Completed	NCT02396342
	AMT-061/Etranacogene dezaparvovec	ssAAV5	Liver-specific	*F9*-Padua	2	Active, not recruiting	NCT03489291
					3	Active, not recruiting	NCT03569891
Spark Therapeutics, Pfizer (Phase 3)	SPK-9001/PF-06838435/Fidanacogene elaparvovec	ssAAV-Spark100	ApoE/hAAT	*F9*-Padua	1/2	Completed	NCT02484092
					2 LTFU *	Recruiting	NCT03307980
					3	Active, not recruiting	NCT03861273
University College, London	FLT180a/Verbrinacogene setparvovec	Synthetic, AAV-S3	Liver-specific	*F9*-Padua	1/2	Terminated (In 2022)	NCT03369444
Freeline Therapeutics							
					1/2	Active, not recruiting	NCT05164471
					1/2 LTFU *	Active, not recruiting	NCT03641703
Baxalta (Shire), Takeda	AskBio009/BAX 335	scAAV8	TTR	*F9*-Padua	1	Active, not recruiting	NCT01687608
Institute of Hematology and Blood Diseases Hospital, China	BBM-H901	Synthetic, AAV843	Liver-specific	*F9*-Padua	1 (12–18- year-old patients)	Not yet recruiting	NCT05709288
					1	Active, not recruiting	NCT04135300
Shanghai Belief-Delivery BioMed Co., Ltd.					1/2	Recruiting	NCT05203679
Institute of Hematology and Blood Diseases Hospital, China	VGB-R04	Synthetic	-	High-specific-activity *F9*	1	Recruiting	NCT05152732
Shanghai Vitalgen BioPharma Co., Ltd.					1/2	Not yet recruiting	NCT05441553
Institute of Hematology and Blood Diseases Hospital, China	ZS801	Synthetic	-	*F9*	-	Not yet recruiting	NCT05630651
					1/2	Not yet recruiting	NCT05641610
St. Jude Children’s Research Hospital	scAAV2/8-LP1-hFIXco	ssAAV2/8	LP1	*F9*	1	Active, not recruiting	NCT00979238
Sangamo Therapeutics	SB-FIX-1501 (ZFN)	ssAAV6	-	-	1	Terminated (In 2022)	NCT02695160
Baxalta (Shire)	SHP648	ssAAV8	-	*F9*-Padua	1/2	Terminated (In 2021)	NCT04394286
Spark Therapeutics	AAV8-hFIX19	ssAAV8	-	*F9*	1	Terminated (In 2019)	NCT01620801
Ultragenyx Pharmaceutical Inc. (previously Dimension Therapeutics)	DTX101	ssAAVrh10	-	*F9*	1/2	Terminated (In 2018)	NCT02618915
					1/2 LTFU	Completed (In 2022)	NCT02971969
Avigen	AAV2-hFIX	ssAAV	hAAT	*F9*	1	Terminated (In 2007)	NCT00076557
SGIMI	YUVA-GT-F901 (Lentivirus)	-	-	-	1	Unknown	NCT03961243

Note: * long-term follow up study.

**Table 2:** Summary of factor IX activity, administered doses, and treatment response for gene therapies with published results.

Therapy and Sponsor	Factor IX Activity	Doses, vg/kg	Patients Responded to Therapy	Reference
AMT-060, UniQure	7.0–7.4% at 4 years	5 × 10^12^, 2 × 10^13^	10/10	[24]
AMT-061/Etranacogene dezaparvovec, UniQure	34.3% ± 4.8% at 18 months	2 × 10^13^	52/54	[19]
SPK-9001/PF-06838435/Fidanacogene elaparvovec, Spark Therapeutics, Pfizer	22.9% ± 9.9% at 5 years	5 × 10^11^	15/15	[25]
FLT180a/Verbrinacogene setparvovec, Freeline Therapeutics	51–78% in 5 patients, 23–43% in 3 patients, 1 patient with 260%	3.84 × 10^11^; 6.4 × 10^11^; 8.32 × 10^11^; 1.28 × 10^12^	9/10, 1 participant continued prophylaxis	[21]
BBM-H901, Institute of Hematology and Blood Diseases Hospital, China	36.9% ± 20.5% at 58 weeks	5 × 10^12^	10/10	[23]
DTX101, Ultragenyx Pharmaceutical Inc (previously Dimension Therapeutics)	5–20% at 8–14 weeks but loss of FIX levels at 32 weeks in all patients except one with ~20% activity	1.6 × 10^12^; 5.0 × 10^12^	6/6	[26]
AskBio009/BAX 335, Baxalta (Shire), Takeda	2.8–58.5% in different dose cohorts at 11 weeks	2 × 10^11^; 1 × 10^12^; 3 × 10^1^	7/8 had measurable factor IX activity up to 11 weeks; only 1/7 had 20% expression for 4 years	[22]
scAAV2/8-LP1-hFIXco, St. Jude Children’s Research Hospital	1–6% at 3.2 years; 5.1 ± 1.7% in a high-dose cohort	2 × 10^11^; 6 × 10^11,^ 2 × 10^12^	6/10, 90% reduction of bleeding episodes and in factor IX prophylaxis use in high-dose cohort	[27,28]
AAV2-FIX, Avigen	Transient at a maximum level of 1.6%	2 × 10^11^ to 1.8 × 10^12^	All 8 participants with a severe form had only local factor IX expression in muscles	[20]

**Table 3:** Advantages and disadvantages of gene transfer and genome-editing approaches.

	Advantages	Disadvantages
Gene transfer	Has been shown to be effective in clinical trials	May not provide a permanent cure, as the transferred gene may not be expressed at high enough levels and may get lost over time
Genome editing	Can be used to permanently correct the specific genetic mutation that causes hemophilia B	Safety concerns and ethical issues of editing the human genome

## Data Availability

Not applicable.
